# Effect of IAPP on the proteome of cultured Rin-5F cells

**DOI:** 10.1186/s12858-018-0099-3

**Published:** 2018-11-12

**Authors:** Samaneh Miraee-Nedjad, Paul F. G. Sims, Jean-Marc Schwartz, Andrew J. Doig

**Affiliations:** 1 Manchester Institute of Biotechnology, The University of Manchester, 131 Princess Street, Manchester, M1 7DN UK; 2 Division of Evolution & Genomic Sciences, Faculty of Biology, Medicine and Health, The University of Manchester, Manchester, M13 9PT UK; 3 Division of Neuroscience and Experimental Psychology, School of Biological Sciences, Faculty of Biology, Medicine and Health, The University of Manchester, Manchester, M13 9PT UK

**Keywords:** Amylin, Type 2 diabetes, Proteomics, Mass spectrometry, Pathway analysis, Protein-protein interactions

## Abstract

**Background:**

Islet amyloid polypeptide (IAPP) or amylin deposits can be found in the islets of type 2 diabetes patients. The peptide is suggested to be involved in the etiology of the disease through formation of amyloid deposits and destruction of β islet cells, though the underlying molecular events leading from IAPP deposition to β cell death are still largely unknown.

**Results:**

We used OFFGEL™ proteomics to study how IAPP exposure affects the proteome of rat pancreatic insulinoma Rin-5F cells. The OFFGEL™ methodology is highly effective at generating quantitative data on hundreds of proteins affected by IAPP, with its accuracy confirmed by In Cell Western and Quantitative Real Time PCR results. Combining data on individual proteins identifies pathways and protein complexes affected by IAPP. IAPP disrupts protein synthesis and degradation, and induces oxidative stress. It causes decreases in protein transport and localization. IAPP disrupts the regulation of ubiquitin-dependent protein degradation and increases catabolic processes. IAPP causes decreases in protein transport and localization, and affects the cytoskeleton, DNA repair and oxidative stress.

**Conclusions:**

Results are consistent with a model where IAPP aggregates overwhelm the ability of a cell to degrade proteins via the ubiquitin system. Ultimately this leads to apoptosis. IAPP aggregates may be also toxic to the cell by causing oxidative stress, leading to DNA damage or by decreasing protein transport. The reversal of any of these effects, perhaps by targeting proteins which alter in response to IAPP, may be beneficial for type II diabetes.

**Electronic supplementary material:**

The online version of this article (10.1186/s12858-018-0099-3) contains supplementary material, which is available to authorized users.

## Background

Type 2 diabetes mellitus, also known as non-insulin-dependent diabetes mellitus (NIDDM), is the most common type of diabetes with more than 285 million people affected worldwide [1]. The disease is characterised by insulin resistance, impaired regulation of hepatic glucose production and β cell dysfunction [2, 3]. Type 2 diabetes is an example of a conformational disease, in which amyloid deposition is likely to be a further contributory factor for pathogenesis [4, 5]. A 37 amino acid peptide, known as islet amyloid polypeptide (IAPP) or amylin, can be isolated from the islets of patients with type 2 diabetes [6]. The normal function of IAPP is to inhibit insulin and glucagon secretion in islets and elsewhere. It affects satiety regulation and inhibits gastric emptying. The peptide may be involved in the etiology of the disease through formation of amyloid deposits and destruction of β islet cells. Further studies have suggested that amyloid deposition contributes to the decreased β cell area and increased β cell apoptosis in human type 2 diabetes [7–9]. Although these studies are proving to be valuable, the exact cytotoxic action of human IAPP and the underlying molecular events leading from IAPP aggregation to β cell death are still largely unknown. The toxic effect of human IAPP is known to involve changes in the expression of a number of genes and proteins [10], though our knowledge of these changes is undoubtedly incomplete. Transcriptional and proteomics studies can therefore facilitate the identification of new genes and gene products that are affected by IAPP.

The development of mass spectrometers with high resolution and high mass accuracy, in combination with different label-free quantitative techniques, has been employed recently to identify new biomarkers for a number of conformational diseases, including type 2 diabetes [11–15]. Li and co-workers studied the serum proteins of diabetic and non-diabetic individuals by label-free quantification and shotgun analysis, and detected expression of 147 proteins, from which 67 and 74 proteins were up- and down-regulated, respectively [16]. Pathway analysis techniques linked these proteins to pathways including lipid metabolism and inflammatory response. Proteome analysis of single pancreatic islets by Waanders and co-workers revealed the significant expression of about 140 proteins, with up-regulation of pathways, including TCA cycle and glycolysis [17]. Analysis of label free LC/MS/MS data by Petyuk and co-workers identified the specific expression of 133 proteins in mouse pancreatic islets [18]. The proteins were correlated to a number of complexes and pathways, including the SNARE complex, which is involved in vesicular trafficking and exocytosis, and the TCA cycle. In another study by Hickey et al. the proteomic analysis of the insulin secretory granules was performed [19]. These cytoplasmic organelles of pancreatic β cells are responsible for the production and secretion of insulin. Their study identified 51 proteins whose main subcellular locations are cytoplasm, mitochondria and endoplasmic reticulum. Some of the proteins identified in this study were: heat shock proteins and protein disulphide-isomerase (both located in endoplasmic reticulum and involved in protein folding), ATP synthase, pyruvate kinase and citrate synthase (all located in mitochondria and involved in energy metabolism), glyceraldehyde-3-phosphate dehydrogenase and aldolase (both located in cytoplasm and involved in energy metabolism) and 14–3-3 zeta isoform (located in cytoplasm and involved in cell signalling). Brunner et al. studied the proteome of the β cells insulin secretory granules using insulin-secreting rat INS-1E cells as a model [11]. They identified the expression of 130 proteins with a majority of the proteins associated with the lysosome.

Schvartz and co-workers found 140 proteins enriched in the mature insulin secretory granule fraction, including: insulin, carboxipeptidase E, PC2, Vamps, secretogranins and chromogranins, vacuolar ATPases and G-proteins involved in exocytosis, members of the v-SNARE complex required for secretion in β-cells, PC1, a key enzyme for proinsulin processing, and exocytosis proteins Noc2 and RhoG [20]. Lim et al. compared effects of IAPP and β-amyloid on human neuroblastoma SH-SY5Y cells and found that the major effect of IAPP was to decrease mitochondrial activity [21].

Overall, these previous studies show that IAPP causes increases of: energy metabolism; vesicle trafficking, secretion and endocytosis; chaperones; and inflammation. This suggests that high levels of IAPP cause cellular stress, increased demand for ATP and enhanced cell signalling.

Protein mass spectrometry is a valuable method for identification and quantitative measurements of many proteins from a complex biological mixture. A tandem mass spectrometry based label free approach, combined with OFFGEL™ fractionation at the protein level [22], was used here to investigate the effects of human IAPP aggregation on the proteome of rat pancreatic insulinoma Rin-5F cells, a cell line widely used in studies of type 2 diabetes. We identified many proteins and pathways whose expressions are affected by IAPP and which are therefore likely to be involved in the pathogenesis of type 2 diabetes.

## Results

### Human IAPP significantly reduces Rin-5F cells viability

**Fig. 1:**
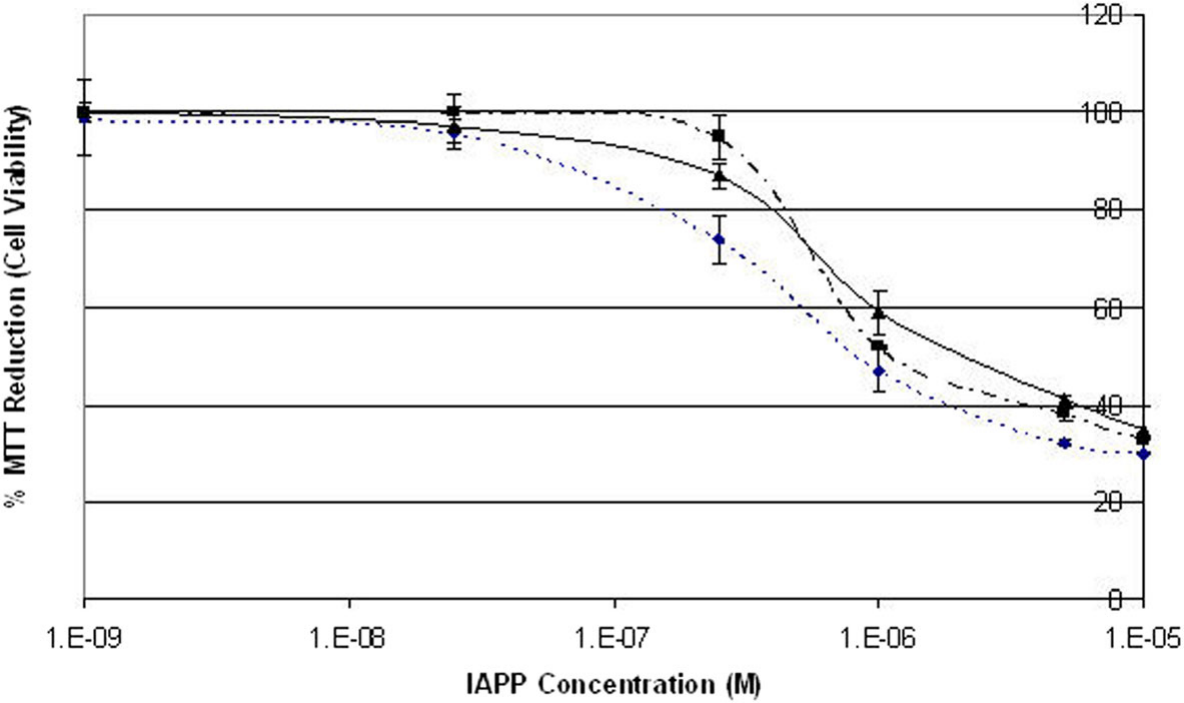
Effects of IAPP cytotoxicity on Rin-5F cells viability. Results for three independent experiments, where the % of MTT reduction shows the Rin-5F cells viability. The viability was calculated by measuring the relative absorbance of the formazan product for Rin-5F cells treated with different concentrations of IAPP, compared to the relative absorbance of the formazan product for live and dead cell controls. Vertical bars indicate standard deviations *n* = 3

**Fig. 2:**
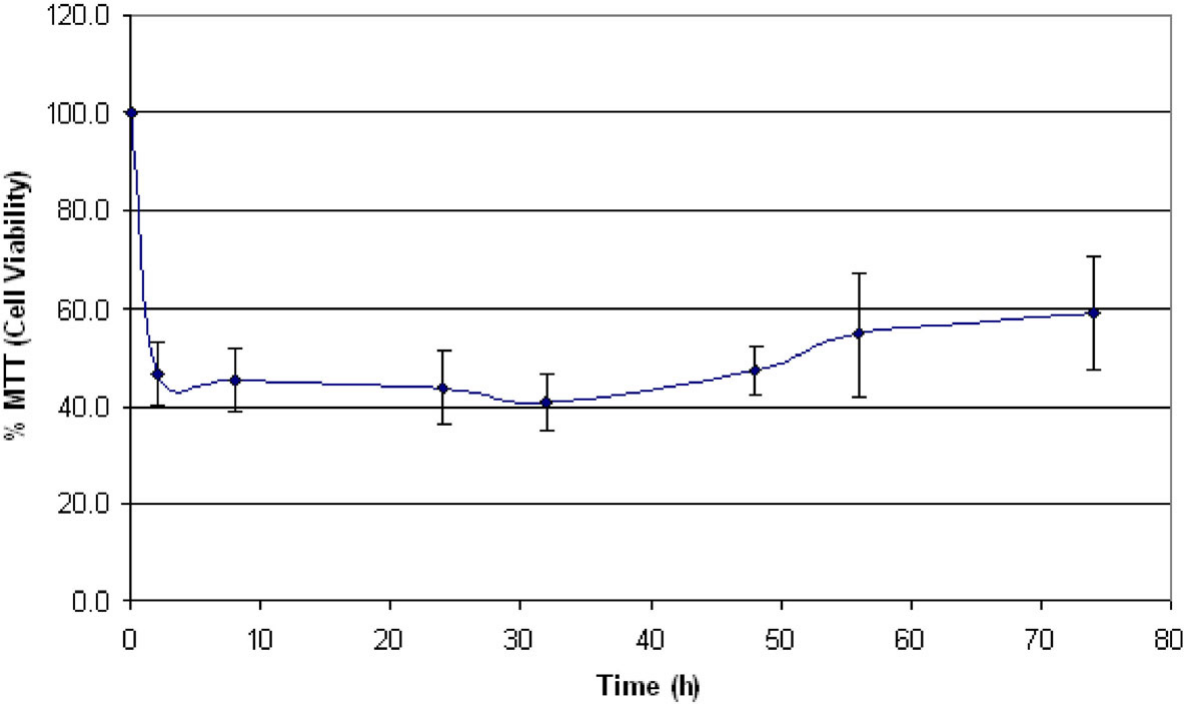
Effects of IAPP cytotoxicity on Rin-5F cells viability at different time points. Viability was calculated by measuring the relative absorbance of the formazan product for Rin-5F cells treated with 5 μM IAPP, compared to the relative absorbance of the formazan product for live and dead cell controls. Vertical bars indicate standard deviations *n* = 3

To investigate the effect of human IAPP on rat Rin-5F cells, MTT assays were carried out, as they reliably report on cell viability via changes in metabolic activity. The cells were treated initially with different concentrations of IAPP (ranging from 10 μM to 1 nM) for 24 h. Monomers and oligomers of IAPP are known to cross the plasma membrane through both endocytotic and non-endocytotic mechanisms in these cells [23]. The viability of Rin-5F cells was reduced with IAPP concentrations from 10 μM to 250 nM (Fig. 1), though there was little change above 5 μM. Addition of DMSO at the same concentrations had no effect on viability (not shown), showing that loss of viability is solely due to IAPP. 5 μM IAPP was therefore used for subsequent work, as the minimal concentration that gives a large change in cell viability. An MTT assay at 5 μM was then carried out to identify the optimal time points at which the IAPP cytotoxicity effect had the largest effect on Rin5F cells’ viability (Fig. 2). The cells’ viability was sharply reduced 2 h after the addition of IAPP and did not change significantly between 2 h to 32 h, after which the MTT signal increased slightly. The 5 μM IAPP concentration with an exposure time of 24 h was therefore used for further experiments. In Rin5F cells, 10 μM IAPP causes less than 20% cell death, whilst 20 μM is required for complete cell death [24]. This conclusion is not dependent on using MTT, as other toxicity assays (e.g. live/dead) show the same result [25]. A concentration of 10 μM is typically used to induce apoptosis [26, 27]. Effects of IAPP on the cells under our conditions will therefore show damaging effects of IAPP before the onset of apoptosis.

### OFFGEL™ electrophoresis successfully separates protein samples from untreated and IAPP treated Rin-5F cells

Prior to OFFGEL™ electrophoresis, proteins from untreated (control) and IAPP treated Rin-5F cells were isolated. Total protein concentrations were calculated using a BSA standard curve (not shown) and equal amounts of protein (about 2 mg) from both samples were fractionated by OFFGEL™ electrophoresis. To determine the fractionation efficiency, one fifth of the protein recovered from each fraction (about 32 μg) was run on an SDS-PAGE gel. The pattern of proteins detected and their band intensities differ significantly between the fractions of each sample, though few differences between untreated and treated Rin-5F cells were apparent by eye (Additional file 1: Figure S1).

### Quantitative analysis of cells by label free tandem MS analysis

The remaining 80% of the proteins recovered from each OFFGEL™ fraction of control and IAPP treated cells were digested into peptides using trypsin and introduced into the tandem mass spectrometer. Table 1 provides a summary of data from three independent experiments, where each experiment was repeated three times. Complete MS results and identified proteins in each OFFGEL™ fraction are in Additional file 2: Table S1. The pI values of the majority of the identified proteins in each fraction were found to be very close to each other (Additional file 2: Table S1), confirming that the OFFGEL™ fractionation of the proteins had been successful. The average pI (pH) values of each fraction rose steadily from Fraction 1 to 12 and were found to be very close to or within the theoretical pH ranges expected of fractions. For some fractions, the calculated pI value differed to some extent from the theoretical pH range, presumably due to post translational modification, since calculated pI values use the unmodified sequences. The percentages of the average coverage of the proteins identified in each fraction were mostly above 25%, adequate for reliable identification. The number of proteins identified in experiment 2 was substantially higher than in experiments 1 and 3 for both control and hIAPP treated cells, though it is unclear why. Many of the proteins identified in experiment 2 are not considered further, since we required confirmation of their identification in at least one additional experiment.

**Table 1:** Proteins Detected in Untreated (Control) and IAPP Treated Rin-5F Cells. Data were obtained from three independent experiments, where each experiment was repeated three times. Data is only reported for proteins detected in all three repeats. F1-F12 are the 12 fractions from OFFGEL™ electrophoresis, separated by pI. Complete data is in Additional file 1: Table S1

Property	F1	F2	F3	F4	F5	F6	F7	F8	F9	F10	F11	F12
Theoretical pH range	< 3.6	3.6–4.2	4.2–4.7	4.7–5.3	5.3–5.9	5.9–6.5	6.5–7.1	7.1–7.6	7.6–8.2	8.2–8.8	8.8–9.2	> 9.2
# proteins control Ex1	29	48	48	16	9	4	53	92	54	26	20	19
Mean pI control Ex1	4.8	4.7	4.9	5.2	5.3	5.8	6.6	7.2	7.6	8.2	8.3	8.6
Mean % protein coverage control Ex1	29.8	32.9	35.9	26.1	31.3	26.6	24.7	26.2	29.5	24.7	27.2	28.1
# proteins IAPP Ex1	33	46	48	53	62	60	70	46	59	15	27	4
Mean pI IAPP Ex1	5.0	4.8	4.9	5.1	5.6	6.2	6.4	7.1	7.5	8.2	8.5	8.8
Mean % protein coverage IAPP Ex1	27.9	33.7	29.6	29.1	25.3	28.1	26.6	25.4	27.4	18.8	31.2	27.4
# proteins control Ex2	106	189	114	180	190	151	214	238	155	192	169	236
Mean pI control Ex2	4.9	4.9	5.1	5.4	6.0	6.4	6.8	7.0	7.6	8.0	8.1	8.5
Mean % protein coverage control Ex2	23.2	29.5	31.8	30.1	29.1	24.2	24.7	24.9	29.5	24.5	26	25.9
# proteins IAPP Ex2	46	131	149	154	152	223	264	270	223	219	145	187
Mean pI IAPP Ex2	4.8	4.8	5.1	5.3	6.0	6.6	6.7	7.1	7.7	8.0	8.6	8.9
Mean % protein coverage IAPP Ex2	21.6	24.3	22.9	26.2	24.5	24.6	24.5	24.2	25.2	24.4	20.7	25.7
# proteins control Ex3	5	57	52	60	45	64	69	75	30	41	46	0
Mean pI control Ex3	4.7	4.8	5.1	5.3	5.9	6.5	6.6	7.2	7.8	7.8	8.5	–
Mean % protein coverage control Ex3	24.2	32.9	29.9	23.7	22.8	26.2	25.3	25.6	27	30.8	31.4	–
# proteins IAPP Ex3	15	33	38	56	50	56	74	53	44	58	27	9
Mean pI IAPP Ex3	5.0	4.8	4.9	5.3	6.0	6.6	6.5	7.0	7.8	7.9	8.4	8.9
Mean % protein coverage IAPP Ex3	25.2	34.8	28.1	30.8	23.7	27.5	23.8	26.3	27.9	32.4	33.5	31.7

### Changes in quantitative protein levels in response to IAPP

To further analyse these data, the quantitative expression patterns of the proteins between the three independent experiments were considered. Our data revealed the common expression of 287 proteins detected in a minimum of two experiments (Additional file 2: Table S1; Additional file 3: Table S2) from which 20 and 5 proteins were found to be significantly down or up-regulated respectively (*p* ≤ 0.05) (Tables 2 and 3). To determine the fold change in the expression level of proteins, the amount of a particular protein in the IAPP treated cells was divided by its corresponding amount in the control cells (IAPP untreated cells). The ratio of every protein was therefore calculated for each experiment and the corresponding ratios of all proteins were then averaged between the three experiments. If a specific protein was not detected in either untreated or IAPP treated samples, the missing value was replaced by the smallest amount that was potentially detected by the machine. In these experiments, the lowest value detected in any sample was 0.1 femtomole for an unknown 35 kD protein (IPI00948374). Proteins were considered to be either up regulated or down-regulated if this ratio was significantly greater than or less than 1, respectively.

**Table 2:** Significantly Down-Regulated Proteins in IAPP Treated Rin-5F Cells. The data were obtained from three independent experiments. To obtain the protein abundance ratio the amount of each protein in the IAPP treated cells was divided by its corresponding amount in the control cells (IAPP untreated cells). The corresponding ratios of all proteins were then averaged between the three experiments. If a specific protein was expressed in either untreated or IAPP treated samples, the missing value was replaced by the smallest amount that was potentially detected by the machine (0.1 femtomole). Differences between IAPP treated and un-treated expression levels are considered to be significant if *p* ≤ 0.05. To determine the *p* value two-tailed Student’s t-test was performed, comparing the three individual control amounts with the three corresponding treated samples

Accession #	UniProt Id	Description	Mean ratio (IAPP treated/control)	SD	*p* Value
IPI00471525	Q68FR9/F1LP72	Uncharacterized protein	0.054	0.076	0.048
IPI00231968	Q5U362	Annexin A4 isoform CRA a	0.097	0.059	0.043
IPI00210357	Q794E4	Heterogeneous nuclear ribonucleoprotein F	0.126	0.040	0.048
IPI00470288	Q9EQS0/P07335	Creatine kinase B type	0.143	0.027	< 0.001
IPI00208215	Q9Z0V6	Thioredoxin dependent peroxide reductase mitochondrial	0.221	0.009	0.034
IPI00387868	O88600/F1LRV4	Heat shock 70 kDa protein 4	0.273	0.021	0.048
IPI00211779	Q63716	Peroxiredoxin 1	0.277	0.001	0.032
IPI00197696	Q0QF43/P04636	Malate dehydrogenase mitochondrial	0.291	0.104	0.013
IPI00201333	D4A0W9	Uncharacterized protein	0.388	0.139	0.050
IPI00190559	Q9EQX9	Ubiquitin conjugating enzyme E2 N	0.453	0.100	0.021
IPI00324893	P63102	14 3 3 protein zeta delta	0.558	0.088	0.001
IPI00200147	P19945	60S acidic ribosomal protein P0	0.678	0.057	0.043
IPI00365935	P83868/B2GV92	Prostaglandin E synthase 3	0.692	0.070	0.005
IPI00200861	P04961	Proliferating cell nuclear antigen	0.877	0.315	0.005
IPI00189925	P54921	Alpha soluble NSF attachment protein	0.005	1.865	0.038
IPI00189989	B2RYK3/P18297	Sepiapterin reductase	0.006	1.089	0.027
IPI00363925	B5DEH4	Uap1l1 protein	0.014	0.234	0.015
IPI00365423	O35511/Q5XI34	Protein phosphatase 2 Formerly 2A regulatory subunit A alpha isoform	0.003	4.101	0.050
IPI00370456	Q4FZT9	26S proteasome non ATPase regulatory subunit 2	0.007	0.491	0.016
IPI00768299	B2GV73/F1LRL8	Actin related protein 2 3 complex subunit 3 Predicted isoform CRA b	0.010	0.798	0.037

**Table 3:** Significantly Up-Regulated Proteins in IAPP Treated Rin-5F Cells. The data were obtained from three independent experiments. To obtain the protein abundance ratio the amount of each protein in the IAPP treated cells was divided by its corresponding amount in the control cells (IAPP untreated cells). The corresponding ratios of all proteins were then averaged between the three experiments. If a specific protein was expressed in either untreated or IAPP treated samples, the missing value was replaced by the smallest amount that was potentially detected by the machine (0.1 femtomole). Differences between IAPP treated and un-treated expression levels are considered to be significant if p ≤ 0.05. To determine the p value two-tailed Student’s t-test was performed, comparing the three individual control amounts with the three corresponding treated samples

Accession #	UniProt Id	Description	Mean ratio (IAPP treated/control)	SD	*p* Value
IPI00194045	Q0QER8/P41562	Isocitrate dehydrogenase NADP cytoplasmic	175	6	0.033
IPI00204532	D3ZYT1	Ubiquitin carboxyl terminal hydrolase	88	3	0.043
IPI00372214	Q4V7C6/D4A7I4	GMP synthase glutamine hydrolyzing	116	0.9	0.035
IPI00421995	Q6MG61	Chloride intracellular channel protein 1	302	1	0.015
IPI00209115	Q6IRH6	Solute carrier family 25 Mitochondrial carrier	87	0.6	0.031

### Down-regulated proteins

Some of the proteins we identified as showing significant responses to IAPP have previously been reported to be linked to type II diabetes:

Heterogeneous nuclear ribonucleoprotein F has been shown to protect against hypertension, renal hypertrophy, and interstitial fibrosis in a diabetic mouse model [28]. Its down-regulation by IAPP may thus lead to diabetes.

Estrogen hormones, such as estradiol-17b, stimulate creatine kinase activity, generating phosphocreatine, a high energy store for brain and muscle. Diabetic rats show a decreased response to estradiol-17b [29]. Similarly, we see a decrease in creatine kinase levels in response to IAPP.

Oxidative stress is an important component of diabetes [30]. Thioredoxin dependent peroxide reductase is used to alleviate oxidative stress by detoxifying reactive oxygen species. Peroxiredoxin 1 similarly reduces hydrogen peroxide. We see that IAPP down-regulates both mitochondrial thioredoxin dependent peroxide reductase and peroxiredoxin 1, thus potentially explaining how oxidative stress is increased in type 2 diabetes. Similarly, loss of functional 14–3-3 protein caused downregulation of thioredoxin reductase in a diabetic mouse model, as well as other adverse effects, such as increases in myocardial apoptosis, cardiac hypertrophy, and fibrosis [31].

Levels of the 70 kDa heat shock protein increase in serum T2D patients [32], presumably due to its cytoprotective chaperone effects. Reduction of chaperone levels by IAPP may thus lead to cytotoxicity.

Diabetes has been shown to lead to alterations in post-translational methylation, phosphorylation and nitration in protein phosphatase 2, resulting in its hyperactivation [33].

Alpha soluble NSF attachment protein is an indispensable component of membrane fusion machinery, required for vesicular transport between the endoplasmic reticulum and the Golgi apparatus. Alterations in its expression are associated with type 2 diabetes [34]. Diabetic rats were shown to have lower levels of Proliferating cell nuclear antigen, a marker of cell proliferation, in rat testicular tissue [35].

Ubiquitin-conjugating enzyme E2E2 (UBE2E2) plays an important role in the synthesis and secretion of insulin. Mutations in UBE2E2 increase risk for type 2 diabetes [36]. 60S acidic ribosomal protein P0 is downregulated, suggesting a decrease in protein synthesis.

### Up-regulated proteins

Reduced cytoplasmic isocitrate dehydrogenase expression in rat insulin secreting cells and isolated rat islet ß-cells resulted in enhanced glucose-induced insulin secretion [37]. The deubiquitining enzyme ubiquitin carboxyl terminal hydrolase is upregulated, again consistent with disruption of protein degradation.

Quantitative RT-PCR miRNA screening in diabetic mice found alteration in the expression of a regulator of the inner mitochondrial membrane phosphate transporter, solute carrier family 25 member 3 (Slc25a3). This provides inorganic phosphate to the mitochondrial matrix and is essential for ATP production [38].

In addition to confirming the roles of the above proteins in type 2 diabetes, we can also report the involvement of various other proteins (Tables 2 and 3).

### **Verification of protein** expression

**Fig. 3:**
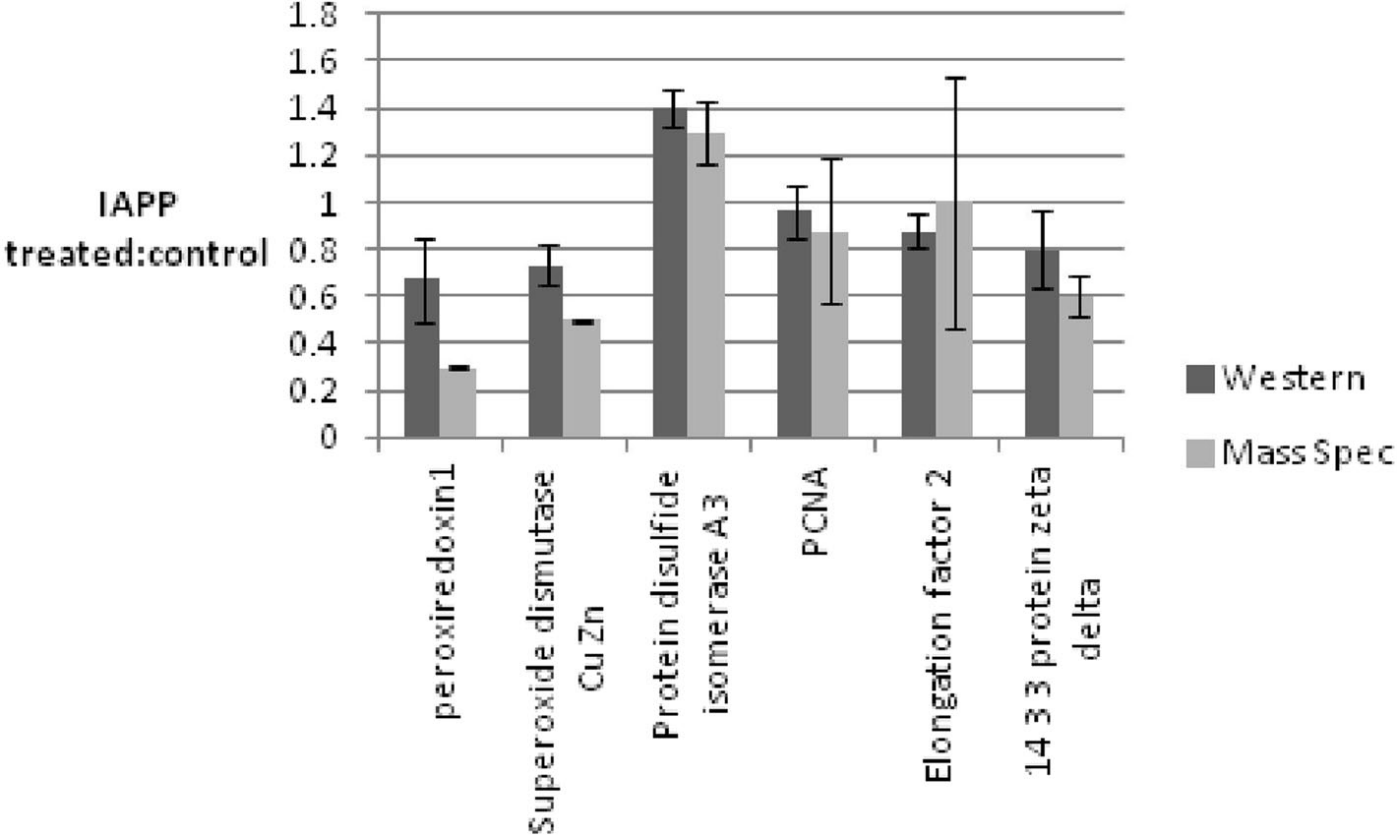
Quantitative analysis of the data obtained from the In Cell Western experiment. The amount of each protein (peroxiredoxin1, Superoxide dismutase Cu Zn, Protein disulfide isomerase A3, PCNA, Elongation factor 2 and 14–3-3 protein zeta delta) expressed in IAPP treated cells was divided by its amount in the control cells. The experiment was repeated three times and the average ratio for each protein was then compared to its corresponding average ratio obtained from the mass spectrometry data. Vertical bars indicate standard deviations *n* = 3

In Cell Western analysis and RT-PCR quantitative techniques were used to confirm the expression of representative proteins identified by tandem MS/MS at the translational and transcriptional levels, respectively. To carry out the In Cell Western analysis, cells were stained for the expression of 6 proteins (peroxiredoxin1, Superoxide dismutase Cu Zn, Protein disulfide isomerase A3, PCNA, Elongation factor 2 and 14–3 -3 protein zeta delta), chosen as they show high, though varying, levels of expression (Additional file 2: Table S1) and have available antibodies. Protein expression was observed in both cultures (Fig. 3), and their changes in levels were consistent with the mass spectrometry data. For example, the expression of peroxiredoxin1 and disulfide isomerase A3 is shown to be reduced and increased, respectively, upon IAPP treatment by both techniques.

**Fig. 4:**
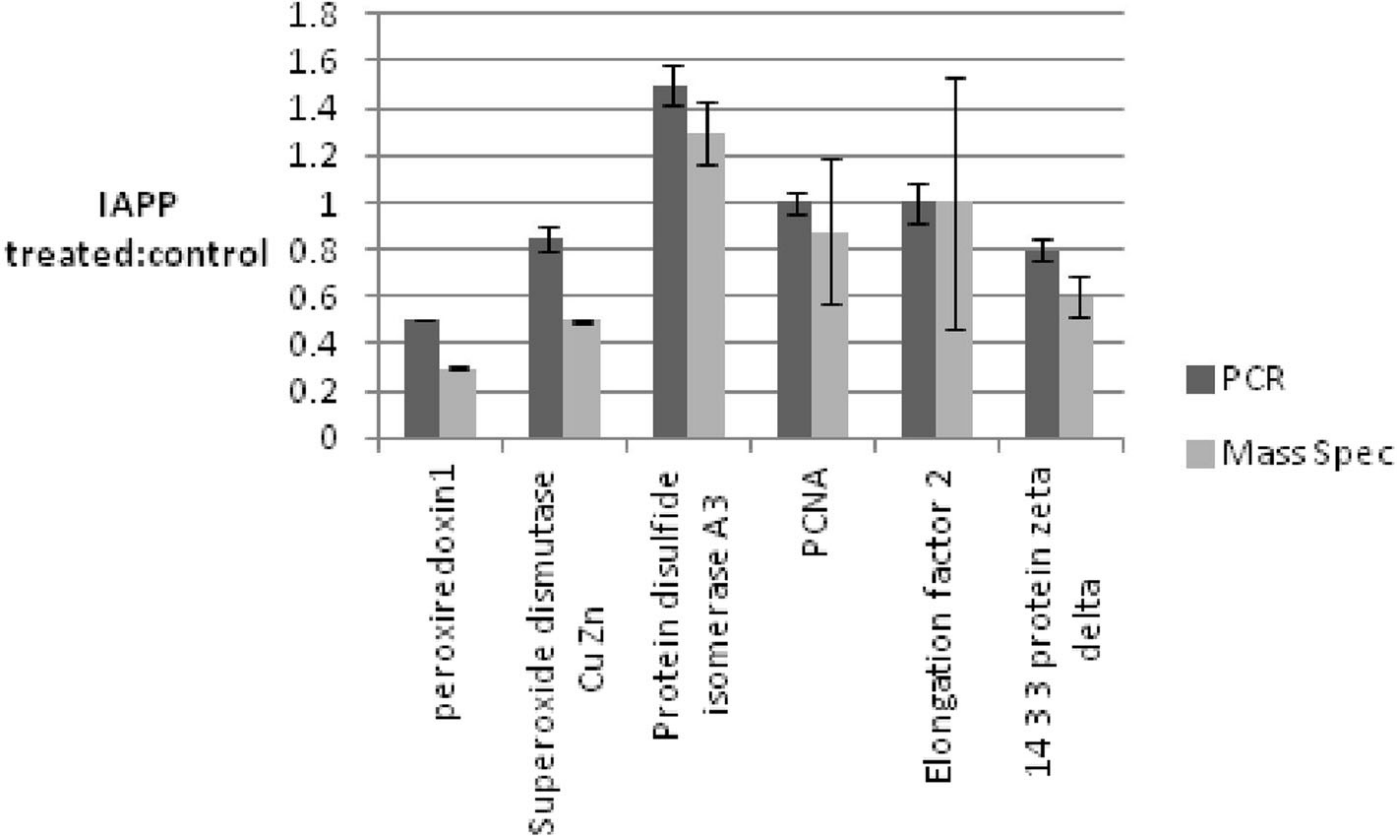
Quantitative analysis of the data obtained from the RT-PCR experiment. The amount of each protein (peroxiredoxin1, Superoxide dismutase Cu Zn, Protein disulfide isomerase A3, PCNA, Elongation factor 2 and 14–3-3 protein zeta delta) was initially normalized against the expression level of the GAPDH housekeeping gene. To obtain the ratio of expression, the amount of each protein expressed in IAPP treated cells was divided by its corresponding amounts in the untreated cells. The experiment was repeated three times. The average ratio for each protein was then compared to its corresponding average ratio obtained from the mass spectrometry data. Vertical bars indicate standard deviations *n* = 3

RT-PCR was also carried out to further confirm the proteomics data, studying the same proteins as in the Western experiment. Rin-5F cells were treated with IAPP for 24 h. RNA was extracted from cells and reverse transcribed before analysis by RT-PCR. Data from three independent experiments showed similar expression of mRNA for all 6 proteins in untreated and IAPP treated cultures to mass spectrometry data (Fig. 4). Perfect agreement between RT-PCR and proteomics data is highly unlikely, since protein abundance is affected by translation and degradation rates, not just expression. Nevertheless, these Western and RT-PCR data do confirm the reliability of our mass spectrometry results.

### Data analysis

To obtain more systematic information about these data, several bioinformatics approaches, including network analysis techniques, pathway analysis and protein complex analysis, were used. This information provides more insight into the function of the proteins, with the aim of facilitating the identification of novel proteins and pathways that might be involved in the pathogenesis of type 2 diabetes.

### Pathway analysis of IAPP responsive proteins

To demonstrate the biological significance of the IAPP responsive proteins, both sets of down- and up-regulated proteins were studied for the Gene Ontology biological process annotations. The number of up-regulated proteins was too small to have significant GO terms associated with it. Four annotations were significant for the down-regulated protein set, namely response to oxidative stress, regulation of cell death, hydrogen peroxide catabolic process and positive regulation of cell repair (Table 4).

**Table 4:** Gene Ontology Analysis for Down-Regulated Proteins. Information is generated using the Gene Ontology analysis tool in DAVID

Gene Ontology	Proteins	Fold Enrichment	*p*-Value
GO:0006979: response to oxidative stress	P04961, Q9Z0V6, Q63716	18	0.011
GO:0010941: regulation of cell death	P63102, Q68FR9	125	0.015
GO:0042744: hydrogen peroxide catabolic process	Q9Z0V6, Q63716	97	0.019
GO:0045739: positive regulation of DNA repair	P04961, Q9EQX9	58	0.032

Analysis using DAVID [39] of down-regulated proteins has further identified several pathways for these proteins. Three significantly down-regulated proteins (P04636, Q9EQS0, B5DEH4) are within the KEGG pathway “rno01130:Biosynthesis of antibiotics”. Other pathways that contain down-regulated proteins are listed in Table 5.

**Table 5:** Pathways Identified for Down-Regulated Proteins

Protein ID	Protein Name	Pathway
P54921	NSF attachment protein alpha(Napa)	Synaptic vesicle cycle
B5DEH4	UDP-N-acetylglucosamine pyrophosphorylase 1 like 1(Uap1l1)	Amino sugar and nucleotide sugar metabolism, Metabolic pathways, Biosynthesis of antibiotics
B2GV73	actin related protein 2/3 complex, subunit 3(Arpc3)	Endocytosis, Fc gamma R-mediated phagocytosis, Regulation of actin cytoskeleton, Bacterial invasion of epithelial cells, Salmonella infection.
P07335	creatine kinase B(Ckb)	Arginine and proline metabolism, Metabolic pathways
Q68FR9	eukaryotic translation elongation factor 1 delta(Eef1d)	Herpes simplex infection
O88600, F1LRV4	heat shock protein family A member 4(Hspa4)	Antigen processing and presentation
P04636, Q0QF43	malate dehydrogenase 2(Mdh2)	Citrate cycle (TCA cycle), Cysteine and methionine metabolism, Pyruvate metabolism, Glyoxylate and dicarboxylate metabolism, Metabolic pathways, Biosynthesis of antibiotics, Carbon metabolism
Q63716	peroxiredoxin 1(Prdx1)	Peroxisome
P04961	proliferating cell nuclear antigen(Pcna)	DNA replication, Base excision repair, Nucleotide excision repair, Mismatch repair, Cell cycle, Hepatitis B, HTLV-I infection
B2GV92, P83868	prostaglandin E synthase 3(Ptges3)	Arachidonic acid metabolism, Metabolic pathways
Q4FZT9	proteasome 26S subunit, non-ATPase 2(Psmd2)	Proteasome, Epstein-Barr virus infection
Q5XI34	protein phosphatase 2 scaffold subunit A alpha(Ppp2r1a)	mRNA surveillance pathway, Sphingolipid signalling pathway, Oocyte meiosis, PI3K-Akt signalling pathway, AMPK signalling pathway, Adrenergic signalling in cardiomyocytes, TGF-beta signalling pathway, Hippo signalling pathway, Tight junction, Dopaminergic synapse, Long-term depression, Chagas disease (American trypanosomiasis), Hepatitis C
P19945	ribosomal protein lateral stalk subunit P0(Rplp0)	Ribosome
P18297, B2RYK3	sepiapterin reductase (7,8-dihydrobiopterin:NADP+ oxidoreductase)(Spr)	Folate biosynthesis, Metabolic pathways
Q9EQS0	transaldolase 1(Taldo1)	Pentose phosphate pathway, Metabolic pathways, Biosynthesis of antibiotics, Carbon metabolism, Biosynthesis of amino acids
P63102	tyrosine 3-monooxygenase/tryptophan 5-monooxygenase activation protein, zeta(Ywhaz)	Cell cycle, Oocyte meiosis, PI3K-Akt signalling pathway, Hippo signalling pathway, Hepatitis B, Epstein-Barr virus infection, Viral carcinogenesis
Q9EQX9	ubiquitin-conjugating enzyme E2N(Ube2n)	Ubiquitin mediated proteolysis

### Analysis of protein-protein interactions

To further evaluate the effect of IAPP on the proteomic profile of the Rin-5F cells, the total set of 287 proteins were analysed for their protein interacting abilities using the STRING database [40]. Out of the 287 target proteins, 156 proteins were found to interact with at least one other protein from the list (Additional file 4: Table S3). These data were used to construct a network for the protein-protein interactions using Cytoscape [41] (Additional file 5: Figure S2). To analyse the proteins within this network, network parameters, such as degree (number of connections between nodes), closeness and betweenness centrality were determined. While closeness centrality determines the centrality of a node that lies inside a particular community (node neighbours from the same group), the betweenness centrality determines the centrality of a node that lies between different communities (node neighbours from different groups) and acts as a bridge to connect these groups together. In other words, the closeness centrality measures the speed of information transfer from a given node to others in a network, while the betweenness centrality indicates how much control a particular node in a network possesses over the interaction of another node in the same network [42].Analysis of the network parameter data for all of the IAPP responsive and un-responsive proteins revealed that the higher degree hub proteins (such as glyceraldehyde 3-phosphate dehydrogenase, ubiquitin A-52 residue ribosomal protein, ATP synthase alpha Subunit 1 and citrate synthase) are positioned mostly in the centre of the protein-protein interaction network. Table 6 summarizes the network parameters data for the top 20 hub proteins with the highest values of degree, closeness and betweenness centrality. As seen in this table, the majority of the proteins (such as triosephosphate isomerase 1, glyceraldehyde-3-phosphate dehydrogenase and ATP synthase) are found in at least two out of the three categories and possess high values for network properties. While many of these proteins are involved in metabolic pathways (such as glutamate dehydrogenase 1 and serine hydroxymethyltransferase 2), others (such as ATP synthase alpha and beta subunits along with ribosomal protein S27a) are involved in other amyloid diseases pathways including Parkinson’s disease, Alzheimer’s disease and Huntington’s disease. Glycolysis (triosephosphate isomerase 1 and phosphoglycerate kinase 1), citrate cycle (citrate synthase and malate dehydrogenase 2) and type II diabetes mellitus (pyruvate kinase) were among the other pathways identified for these proteins.

**Table 6:** Twenty Top Proteins for Protein-Protein Interaction Network Parameter Terms (network degree, closeness centrality and betweenness centrality). Proteins with a closeness centrality of 1.0 were excluded as they correspond to isolated protein pairs

Protein	Accession #	Degree	Fold change	Protein	Accession #	Closeness centrality	Fold change	Protein	Accession #	Between-ness centrality	Fold change
Tpi1	IPI00231767	47	1.8	Gapdh	IPI00555252	0.481	0.34	Gapdh	IPI00555252	0.135	0.34
Mdh2	IPI00197696	38	0.29	Tpi1	IPI00231767	0.463	1.5	Pcna	IPI00200861	0.127	0.89
Sod2	IPI00211593	38	1.1	Alb	IPI00191737	0.460	1.3	Actb	IPI00189819	0.0683	0.94
Gapdh	IPI00555252	37	0.34	Pcna	IPI00200861	0.444	0.88	Alb	IPI00191737	0.0645	1.3
Cs	IPI00206977	35	1.1	Atp5a1	IPI00396910	0.441	1.1	Eef2	IPI00203214	0.0574	1.1
Atp5a1	IPI00396910	34	1.1	Atp5b	IPI00551812	0.440	0.44	Atp5a1	IPI00396910	0.0522	1.1
Atp5b	IPI00551812	33	0.44	Pgk1	IPI00231426	0.438	0.92	Tpi1	IPI00231767	0.0506	1.5
Pkm2	IPI00231929	31	0.59	Eef2	IPI00203214	0.436	1.1	Cfl1	IPI00327144	0.0387	1.4
Shmt2	IPI00195109	30	1.2	Shmt2	IPI00195109	0.436	1.2	Glud1	IPI00324633	0.0383	2.1
Glud1	IPI00324633	30	2.1	Cs	IPI00206977	0.435	1.1	Gnb2l1	IPI00231134	0.0372	1.5
Dld	IPI00365545	30	1.9	Pkm2	IPI00231929	0.431	0.59	Gmps	IPI00372214	0.0371	120
Alb	IPI00191737	29	1.3	Dhfr	IPI00200419	0.429	1.2	Rad23b	IPI00210495	0.0359	0.96
Pgk1	IPI00231426	27	0.92	Mdh2	IPI00197696	0.424	0.29	Stip1	IPI00213013	0.0343	2.0
Pcna	IPI00200861	25	0.88	Actb	IPI00189819	0.423	0.94	Mdh2	IPI00197696	0.0332	0.29
Gmps	IPI00372214	25	120	Hspa4	IPI00387868	0.423	0.27	Tubb5	IPI00197579	0.0317	0.15
Eef2	IPI00203214	24	1.1	Sod2	IPI00211593	0.422	1.1	Shmt2	IPI00195109	0.0303	1.2
Mdh1	IPI00198717	24	6.0	Gmps	IPI00372214	0.419	120	Eif5a	IPI00211216	0.0286	0.34
Gnb2l1	IPI00231134	23	1.5	Gnb2|1	IPI00231134	0.413	1.5	Dars	IPI00206224	0.0257	1.1
Sod1	IPI00231643	23	0.52	Txn1	IPI00216298	0.411	29	Aprt	IPI00950965	0.0256	0.016
Txn1	IPI00231368	23	29	Sod1	IPI00231643	0.409	0.52	Ywhaz	IPI00324893	0.0250	0.56

Most of the identified hub proteins in the network were unresponsive to the effect of IAPP. As seen in Table 7, many of the up and down regulated proteins in this network are not strong hubs and they are not highly connected to other proteins in the network. In other words, the analysis of the network parameter data revealed a very weak correlation between the fold change and connectivity or centrality. This finding is in agreement with previous reports which showed that about 78% of genes/proteins implicated in diseases are found to be non-essential. As hub proteins are more likely to be encoded by essential genes, disease genes do not tend to correlate with hubs [43, 44]. Our data suggests that there is only a weak tendency for IAPP responsive proteins to be associated with hubs.

**Table 7:** Network Parameter Data (Degree, Closeness Centrality and Betweenness Centrality) for the Up- and Down-Regulated Proteins in the Protein-Protein Interaction Network

Protein Ids	Fold Change	Closeness Centrality	Degree	Betweenness Centrality
Mdh2 / P04636	0.29	0.424	38	0.0332
Hspa4 / F1LRV4	0.27	0.423	16	0.0224
Ywhaz / P63102	0.56	0.369	10	0.0251
Arbp / P19945	0.68	0.388	13	0.0142
Ube2n / Q9EQX9	0.45	0.248	1	0.0
Slc25a3 / Q6IRH6	87	0.364	11	0.0011
Prdx3 / Q9Z0V6	0.22	0.321	5	0.0014
Idh1 / P41562	175	0.338	9	0.0
Ppp2r1a / O35511	0.003	0.350	7	0.0093
Ptges3 / B2GV92	0.69	0.330	3	0.0003
Eef1d / F1LP72	0.054	0.312	4	0.0
Pcna / P04961	0.88	0.444	25	0.127

The effect of IAPP on the protein complexes was also investigated using the MIPS database (Mammalian Protein-Protein Interaction Database) [45]. Proteins up or down-regulated by IAPP treatment were submitted to MIPS. Six protein complexes were down-regulated and none were up-regulated (Table 8). To determine the fold changes in the expression level of protein complexes the number of affected subunits within each complex in the IAPP treated cells was divided by its corresponding amount in the control cells. The Alpha soluble NSF attachment protein was found in four complexes, three of which are SNARE complexes, while two proteins were found in the CLIC4 complex. The SNARE complex is involved in vesicular trafficking and exocytosis, and the TCA cycle, and has been linked to type 2 diabetes by several other groups [18, 20]. CLIC4 is a chloride channel involved in stabilisation of cell membrane potential, transport, maintenance of intracellular pH and regulation of cell volume.

**Table 8:** Protein Complexes Identified for Down-Regulated Proteins using MIPS

Complex name	Protein Id	Description	Average ratio of subunits within the complex (IAPP/Control	Average standard deviations	Mean *p*-Value
CLIC4 complex	P63102	14 3 3 protein zeta delta	0.35	0.06	0.001
CLIC4 complex	P07335	Creatine kinase B type			
SNARE complex Snap25	P54921	Alpha soluble NSF attachment protein	0.0045	1.87	0.038
SNARE complex Stx1a	P54921	Alpha soluble NSF attachment protein	0.0045	1.87	0.038
SNARE complex Stx4	P54921	Alpha soluble NSF attachment protein	0.0045	1.87	0.038
Nsf-Stx1a-Napa complex	P54921	Alpha soluble NSF attachment protein	0.0045	1.87	0.038

## Discussion

Our data analysis has identified pathways and protein complexes that have been affected by toxic (though not lethal) levels of IAPP to Rin-5F cells and which may be involved in the pathogenesis of type II diabetes. IAPP added to Rin-5F cells provides a simpler, more homogeneous model than, say, post-mortem islets cells from a diabetic patient, and allow us to study early cellular events caused by a toxic peptide. The strongest effect of the addition of IAPP is disruption of protein synthesis and degradation, together with induction of oxidative stress. This agrees well with the work of Casas et al., who found that impairment of the ubiquitin-proteasome pathway is implicated in ER stress–mediated pancreatic β-cell apoptosis [46]. Oxidative stress is known to be an important component of diabetes [30] IAPP also induces decreases in protein transport and localization. Most of the pathways that we find to be affected differ from previous proteomic work on IAPP, though we do see effects on TCA Cycle, heat shock and cell signaling.

## Conclusions

The OFFGEL™/HI3 methodology is highly effective at generating quantitative data on hundreds of proteins affected by toxic IAPP. Its accuracy is confirmed by In Cell Western and Quantitative Real Time PCR results. Our results are consistent with a model where IAPP aggregates overwhelm the ability of a cell to degrade proteins via the ubiquitin system, leading to DNA damage, decreases in protein transport and ultimately apoptosis.

## Methods

### Cell culture

The rat pancreatic insulinoma Rin-5F cell line was purchased from the European Collection of Cell Cultures (*ECACC,* Wiltshire, UK). The cells were cultured in RPMI 1640 medium supplemented with 10% (*v*/v) Foetal Bovine Serum (FBS) (PAA Laboratories, UK), and 2 mM Glutamine. The cells were maintained in a 5.0% CO_2_ humidified atmosphere at 37 °C.

### hIAPP cytotoxicity

hIAPP was purchased from Bachem (Germany). The powder was dissolved in high-grade 1,1,1,3,3,3-hexafluoroisopropanol (HFIP) (Sigma, UK) to a stock concentration of 1 mM. Reconstituted hIAPP was snap-frozen in liquid nitrogen and freeze dried to remove HFIP. Freeze dried hIAPP was kept at − 20 °C and dissolved in dimethyl sulfoxide (DMSO) (Sigma, UK) to the required concentration before each experiment. The cytotoxicity of IAPP on Rin-5F cells was assessed by the MTT assay [47] according to the manufacturer’s protocol (Sigma, UK). The cells were initially plated in triplicate at a density of 2.5 × 10^4^ cells/well in 96 well plates in Optimal media (Invitrogen, UK) supplemented with 5% (*v*/v) Foetal Bovine Serum (FBS) (PAA Laboratories, UK), 2 mM Glutamine and non-essential amino acids (PAA Laboratories, UK). After overnight growth, IAPP was added to the cells to give the required concentration. Plates were further incubated for the times indicated in a 5% (v/v) CO_2_ and 95% (v/v) air incubator. For the dead cell controls, 0.5% (v/v) Triton X-100 (Sigma-UK) was added to the wells. Live cell controls contained Rin-5F cells only. The results of the MTT assays are expressed as percentage of MTT reduction (percentage of cell viability) and calculated as: % MTT reduction (% cell viability) = {(C-A) ÷ (B-A)} × 100%, where C is the mean absorbance of the cells treated with IAPP (*n* = 3), A is the mean absorbance of the dead cell control samples (*n* = 3) and B is the mean absorbance of the live controls samples (*n* = 3).

### Protein fractionation and identification

A urea/thiourea extraction was utilized to extract the proteins from 70 to 80% confluent T75 flasks (about 10^7^ cells) of untreated or 5 μM IAPP treated Rin-5F cells. Rin-5F cell pellets were extracted in 1 ml of lysis buffer containing 9.5 M urea, 2 M thiourea, 4% (*w*/*v*) CHAPS, 1% (w/v) DTT, 2.5 mM EDTA and 2.5 mM EGTA (all from Sigma-UK). Samples were vortexed 5 times for 10 s each time and then left at room temperature for 30 min. Cell extracts were then centrifuged at 5000 g for 10 min. The supernatant was collected and, prior to their fractionation by OFFGEL™, 4 times their volume of ice cold acetone was added. Samples were kept at − 20 °C for 1 h and were then centrifuged at 5000 g for 10 min. The pellet was air dried at room temperature and was kept at − 20 °C for further analysis.

The OffGEL™ system (Agilent 3100 OFFGEL™ fractionator, Agilent Technologies) utilises a 12-well chamber in which is placed on an immobilized pH gradient gel. The protein solution is introduced into the open top of each of the chambers. An electric field is applied through the chamber which facilitates the migration of charged proteins out of the chamber, into the gel, and from one well to another until they reach the well where the pH of the gel is equal to the pI of the protein. The proteins can be then recovered in solution, acetone precipitated and used for further analysis by tandem mass spectrometry, as described below.

The acetone precipitated cell pellets destined for OFFGEL™ fractionation were dissolved in sample buffer (7 M Urea, 2 M thiourea 1% DTT, 10% glycerol (all from Sigma-UK) and 1.0% (*v*/v) IPG buffer pH 3–10 (GE Healthcare, UK)). Protein concentrations in all samples were measured using a 2-D Quant Kit (Amersham Biosciences, UK) with a standard curve using Bovine serum albumin (BSA). For each sample, 150 μl (170 μg) was loaded into each of the twelve wells on the OFFGEL™ fractionator. Fractionation was carried out using a program optimized to focus samples for about 24 h over which the voltage was gradually increased from 500 V to 1000 V before a final limiting voltage of 8000 V was applied. A maximum current of 50 μA was applied throughout the focussing stage and the temperature was stabilised to 22 °C during the fractionation. The protein fractions were recovered from each well at the end of run. About 30 μl of the sample (20%of the total) recovered from each OFFGEL™ fraction was placed in a separate tube for analysis by SDS-PAGE gel electrophoresis and the rest was utilised for liquid chromatography tandem MS analysis experiments. The two samples obtained from each fraction were then acetone precipitated separately. The procedure was as described above, but the samples were first diluted with two volumes of distilled water before addition of the acetone.

The efficiency of the OFFGEL™ separation was monitored using 1D electrophoretic analysis. To this end, the acetone precipitates derived from the smaller samples recovered from each fraction were reconstituted in 20 μl of loading buffer (100 mM Tris pH 6.8, 4% SDS, 20% glycerol, 1% *v*/v β-mercaptoethanol and 0.1% bromophenol blue). The samples and molecular weight marker (PageRuler™, Fermentas, UK) were heated at 100 °C for 10 min and then applied to a 10% resolving gel (using the Bio-Rad Protean II XL system) for protein electrophoresis. The gels were run at 50 V through the stacking gel and 150 V through the resolving gel. Proteins were stained using 0.5% Coomassie Brilliant Blue G250 (Sigma, UK), 40% ethanol and 10% acetic acid for 1 h, and destained in 20% ethanol and 10% acetic acid for 2 h.

To digest the proteins and isolate the resulting tryptic peptides generated from OFFGEL™ fractionation, the larger of the acetone precipitated pellets obtained from each OFFGEL™ fraction were reconstituted in 150 μl of 1X digestion buffer containing 1 M ammonium bicarbonate, 1 M CaCl2 and 1.2 g urea (all from Sigma, UK). The samples were then placed in a 10 kDa filter (Ambion 0.5 ml, 10,000 MW cut- off centrifugal filter, Millipore UK Limited, UK) and centrifuged at 14000 g for 15 min. The filtrate was removed from the filter holder and 2.5 μl of 10 mM DTT was added to the filter. The tubes were incubated at 37 °C for 20 min and 2 μl of 30 mM iodoacetamide (Sigma-UK) was then added to the filter. The tubes were incubated at room temperature for 20 min. At this point 200 μl of 1X digestion buffer was added to the tubes which were centrifuged at 14000 g for 15 min. The filtrate was removed from the filter holder and 5 μl of 0.1μg/μl of trypsin (Roche Diagnostics, UK) was added to the filters. The tubes were then incubated at 37 °C overnight. To stop the reaction, 1 μl of formic acid and 200 μl of 50 mM ammonium bicarbonate were then added to the filters. The tubes were then centrifuged at 14000 g for 15 min. The filtrate was retained and vacuum centrifuged until all solution was removed. The dried pellets were reconstituted in 40 μl of buffer A, containing 0.1% formic acid and 10% acetonitrile (all from Sigma, UK), and kept at 4 °C for further analysis.

For the label free mass spectrometric quantitative analysis of the samples, 7.5 μl of the tryptic digest from each fraction was mixed with 5 μl of the rabbit glycogen phosphorylase B standard tryptic digest at 50 femtomoles μL^− 1^, (Waters, UK). A 2.5 μL aliquot of the mixture of sample and standard digests was then injected three times into the mass spectrometer. HPLC separation of tryptic peptides was carried out using a Waters nanoACQUITY™ UPLC fitted with a Symmetry® C18 HPLC trapping column of 20 mm length and an internal diameter (ID) of 180 μm (Waters, Ltd). Sample loading time was 1 min at a flow rate of 15 μl min^− 1^ in 97% water, 3% acetonitrile. The trapped peptides were then eluted on to a BEH130 C18 HPLC analytical column of 25 cm length and an ID of 75 μm with an elution gradient of 3–40% acetonitrile in water (containing a constant 0.1% formic acid) running for 30 min at a flow rate of 300nLmin^− 1^. The column temperature was maintained at 35 °C and peptides were eluted via a 10 μm PicoTip emitter (New Objective). into the nano-ESI source of a Waters Synapt G1 High Definition instrument controlled using MassLynx v4.1 (Waters Ltd.). Immediately before analysis, the mass spectrometer was calibrated using the product ion spectrum of glu-fibrinopeptide B (500fmol μl^− 1^ of peptide in 50% water, 50% acetonitrile containing 0.1% formic acid). The instrument was operated in V mode using data independent (MS^E^) acquisition. The low energy, survey, scan was performed between *m*/*z* 50–2000 with a trap cell collision energy of 6 eV. The elevated energy, product ion, scan was acquired similarly except that the trap collision energy was ramped from 15 to 40 eV during data acquisition. Transfer cell collision energy was 4 eV for both scans and the lock mass was recorded every 30 s. After data-independent acquisition, protein identification was carried out using the UniProt/Swiss-Prot database (Release 2012_04) and a search algorithm embedded within the ProteinLynx Global Server software package, (version 2.4, Waters Ltd.) which was specifically developed for the qualitative identification of proteins over a wide dynamic range in complex biological samples [48]**.** The following settings were applied; automatic settings for precursor and product ion mass tolerance; minimum fragment ion matches per peptide, 8; minimum fragment ion matches per protein, 15; minimum peptide matches per protein, 1; fixed modification, carbamidomethyl Cys; variable modification, oxidised Met; number of missed cleavages, 1; false positive rate, 1%.

### Protein quantification strategy

Proteins were quantitated using a HI3 label-free approach that compares the intensity of the precursor ions identified from sample proteins with those derived from a standard present at known concentration [49]. The algorithm used, also embedded within the ProtynLynx Global Sever software package, integrates the volume of each extracted ion (charge state reduced, deisotoped and mass corrected) across the mass chromatogram. Protein concentrations are estimated by comparison of the average intensity of the three most abundant peptides, from a particular protein released from the chromatography columns, with the equivalent value determined for a known amount of the internal standard (a tryptic digest of rabbit phosphorylase B) introduced to the experimental samples before analysis. Each of the 12 OFFGEL™ fractions derived from a given sample were analysed separately and the data were then combined to give the total amount of a given protein present in that sample. Each experiment was conducted on three separate occasions and each of these biological replicates was analysed three times. Changes in expression levels were only considered for those proteins detected and quantitated in a minimum of two of the three biological replicates.

### Quantitative real time PCR

RNA was extracted from 70 to 80% confluent T75 flasks (about 10^7^ cells) of untreated and 5 μM IAPP treated Rin-5F cells using an RNeasy kit (Qiagen, West Sussex, UK) according to the manufacturer’s instructions. The RNA concentration and purity were measured using an Agilent 2100 Bioanalyser. The RNA purity was measured from the A260nm/A280nm ratio and was always in the range of 1.9 to 2.0. RNA was normalized for all the cell samples to 8.5 μg for the cDNA synthesis and reverse transcribed using qScript® cDNA SuperMix (Quanta Biosciences, Gaithersburg, MD, U.S.A) according to the manufacturer’s instructions. Quantitative real time PCR was performed using the Light-Cycler® 480 II platform (Roche Diagnostics, UK). The PCR was performed in 10 μl of reaction volume with 5 μl of qPCR MasterMix Plus for SYBR® Green, 4 μl of 10x diluted cDNA and 0.1 μl of each forward and reverse primer at the stock concentration of 20 μM. The mixtures were then loaded in a 384 well plate. The plates were sealed with Microseal ‘B’ film (Bio-Rad, UK) and centrifuged at 800 g for 1 min. The PCR conditions were: activation of enzymes at 95 °C for 10 min, 40 cycles of denaturation at 95 °C for 15 s, annealing and extension at 60 C for 1 min.

### In cell Western analysis

The cells were initially plated in triplicate at a density of 2.5 × 10^4^ cells/well in 96 well plates. For the treated samples, hIAPP was added to the cells to final concentrations of 5 μM per well. Plates were incubated for 24 h at 37 °C. The In Cell Western assay was then carried out using In-Cell Western™ Kit II (LI-COR Biosciences, UK) according to the manufacturer’s instructions. Primary antibodies for this assay were used as follows: Anti-PCNA antibody, 1:100; Anti-EEF2 antibody, 1:120; Anti-Superoxide Dismutase 1 antibody, 1:200; Anti-Peroxiredoxin1 antibody, 1:250; Anti-ERp57 antibody,1:500; Anti-14-3-3 zeta antibody 1:500 (all Abcam-UK). Donkey anti-rabbit-IRDye 700CW (LI-COR Biosciences, UK) secondary antibody was diluted 500-fold and used for the detection of the primary antibodies. The Odyssey Infrared Imaging System (LI-COR Biosciences, UK) was then used to scan the plate and to detect the signal from the secondary antibody in the 800 nm channel.

### Data analysis

KEGG pathways and GO gene ontology research tool annotations of the identified proteins were obtained using the DAVID Web-based tool [39]. Protein-protein interactions were determined by querying each of the proteins detected and quantitated in a minimum of two replicates using the STRING database [40]. The data obtained from the STRING database were imported into the Cytoscape software to construct a network of protein-protein interactions [41]. The NetworkAnalyzer Cytoscape plugin was used to analyse the interaction network including degree [50], closeness centrality [42] and betweenness centrality [51]. The MIPS database [45] was used to investigate the protein complexes that contain the identified proteins.

Numerical data were subject to statistical analysis using standard deviation and Student’s t-test.

## Additional files

Additional file 1: **Figure S1.** SDS-PAGE analysis of the OFFGELTM fractions of untreated and IAPP treated Rin-5F cells. (DOCX 846 kb)

Additional file 2: **Table S1.** Protein Mass Spectrometry Data. (ZIP 2439 kb)

Additional file 3: **Table S2.** Protein Mass Spectrometry Data Table Descriptors. (DOCX 12 kb)

Additional file 4: **Table S3.** Protein-Protein Interactions. (XLSX 44 kb)

Additional file 5: **Figure S2.** Protein-Protein Interaction Network; Up-regulated proteins are colour coded by red, down-regulated by green and unchanged by yellow. (TIF 877 kb)

## Abbreviations

BSA: Bovine serum albumin
CHAPS: 3-[(3-Cholamidopropyl)dimethylammonio]-1-propanesulfonate
DMSO: Dimethylsulphoxide
DTT: Dithiothreitol
EDTA: 2,2′,2″,2‴-(Ethane-1,2-diyldinitrilo)tetraacetic acid
EGTA: Ethylene glycol-bis(2-aminoethylether)-*N*,*N*,*N′*,*N′*-tetraacetic acid
ER: Endoplasmic reticulum
GO: Gene ontology
HFIP: 1,1,1,3,3,3-Hexafluoroisopropanol
hIAPP: Human islet amyloid polypeptide
IAPP: Islet amyloid polypeptide
IPG: Immobilized pH gradient
LC: Liquid chromatography
MS: Mass spectrometry
MTT: 3-(4,5-Dimethylthiazol-2-yl)-2,5-diphenyltetrazolium bromide
NIDDM: Non-insulin-dependent diabetes mellitus
PCR: Polymerase chain reaction
T2D: Type 2 diabetes
TCA: Tricarboxylic acid
UBE2E2: Ubiquitin-conjugating enzyme E2E2
