# Supplementary material for: Effect of IAPP on the proteome of cultured Rin-5F cells
Source: BMC Biochem. 2018 Nov 12;19:9. doi: 10.1186/s12858-018-0099-3 (PMC6233276; doi:10.1186/s12858-018-0099-3)
Supplement: Supplementary file 1 — Figure S1. SDS-PAGE analysis of the OFFGELTM fractions of untreated and IAPP treated Rin-5F cells. (DOCX 846 kb) [file 12858_2018_99_MOESM1_ESM.docx]

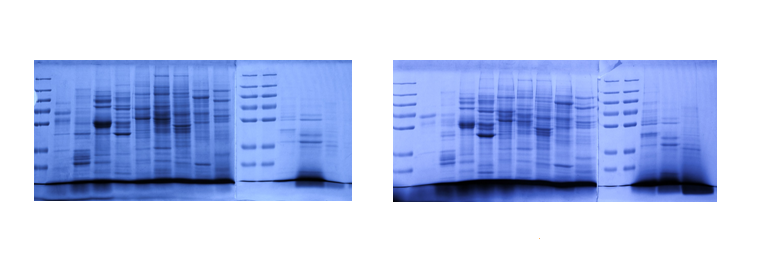


a

Ex1

m 1 2 3 4 5 6 7 8 9 m m 10 11 12

m 1 2 3 4 5 6 7 8 9 m m 10 11 12

IAPP treated Rin-5F cells

Untreated Rin-5F cells (control)

Ex2

b


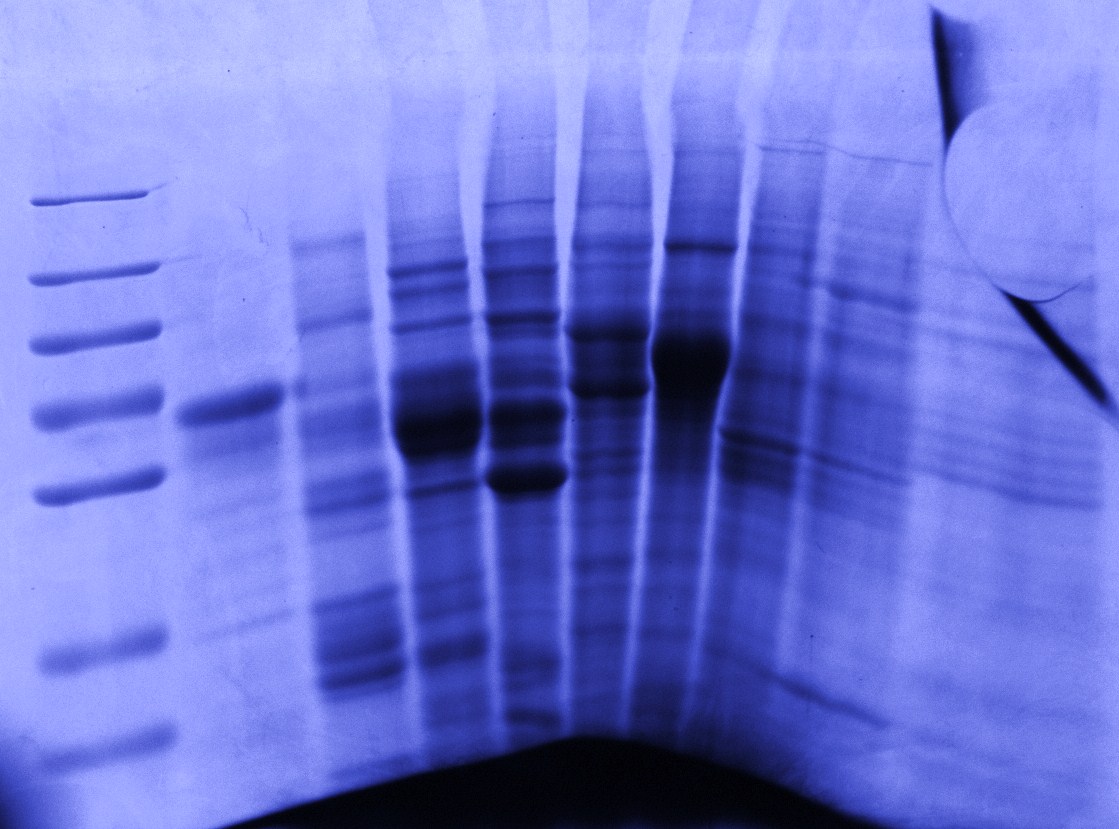

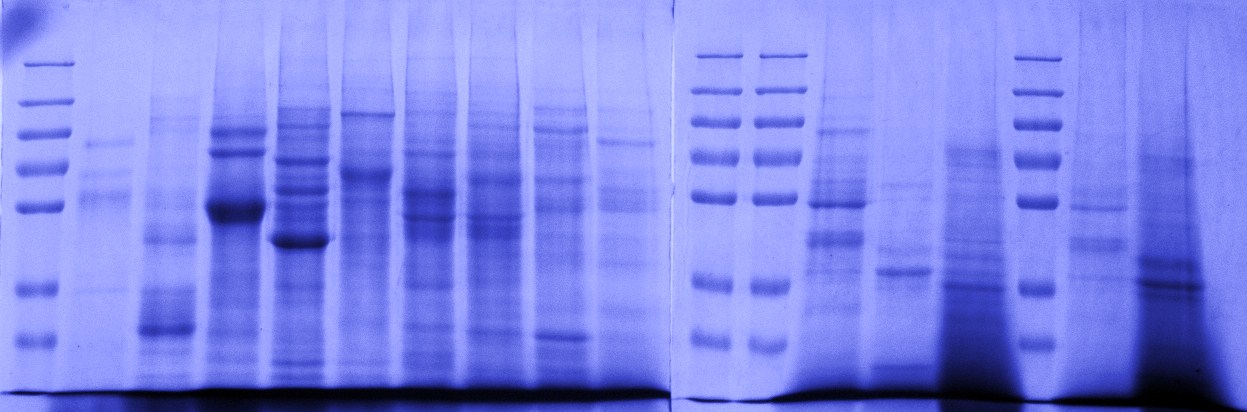

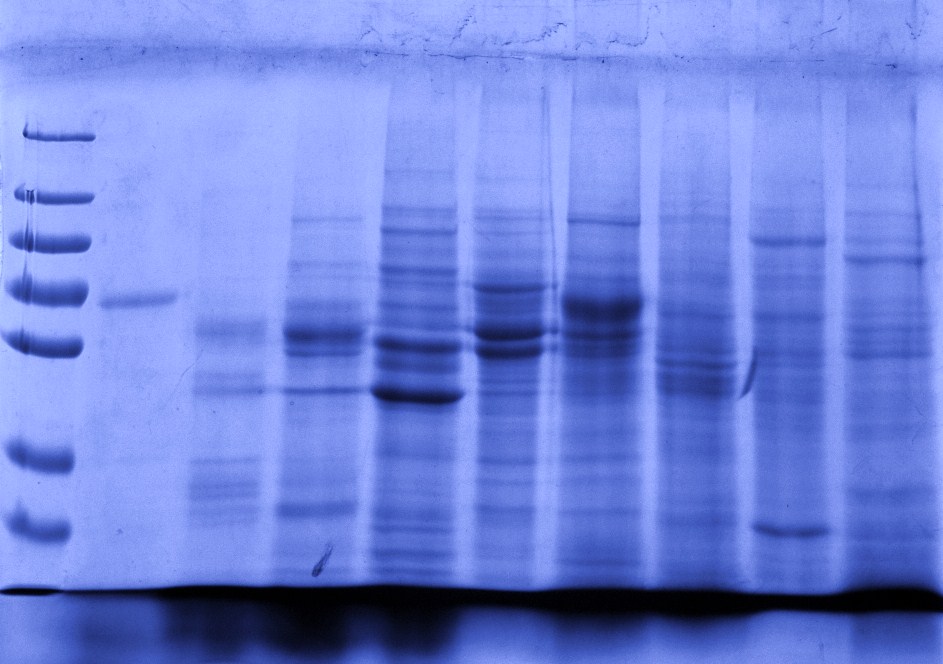

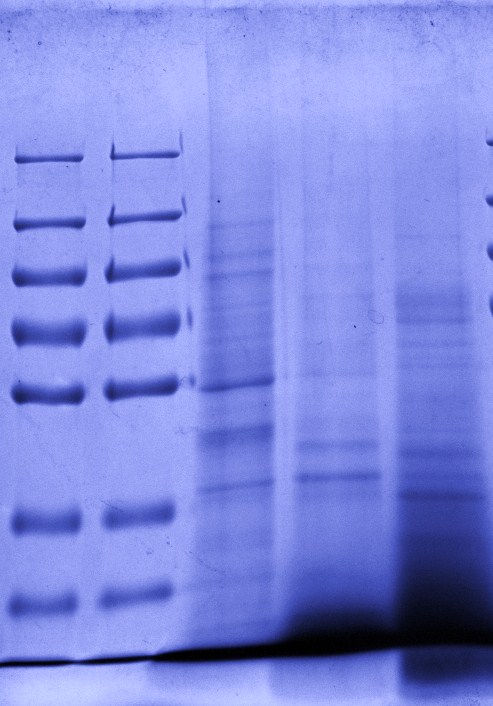


m 1 2 3 4 5 6 7 8 9 m m 10 11 12

m 1 2 3 4 5 6 7 8 9 m m 10 11 12

IAPP treated Rin-5F cells

Untreated Rin-5F cells (control)

Ex3

c


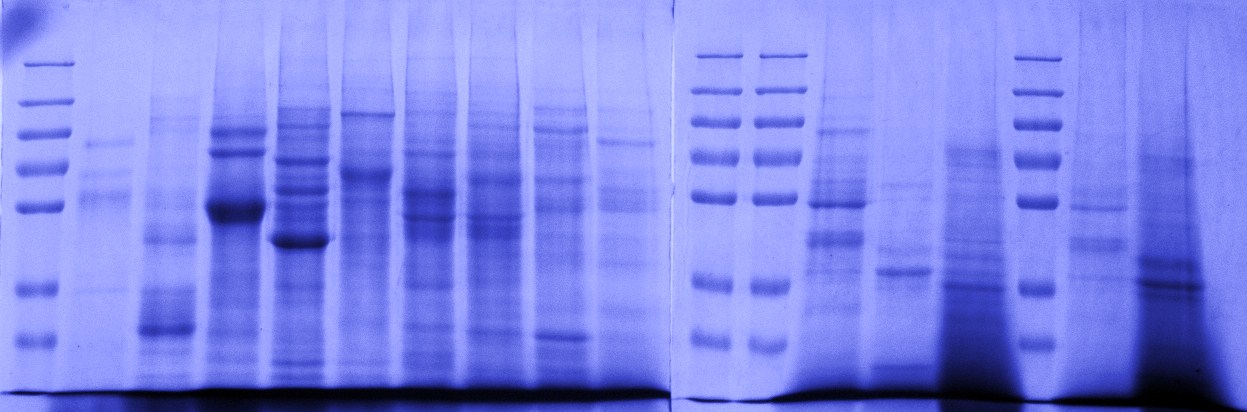

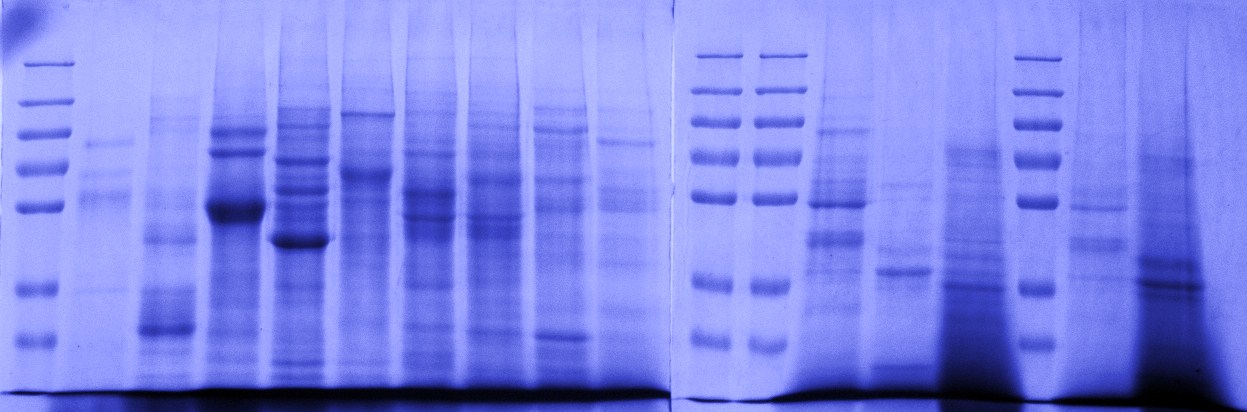

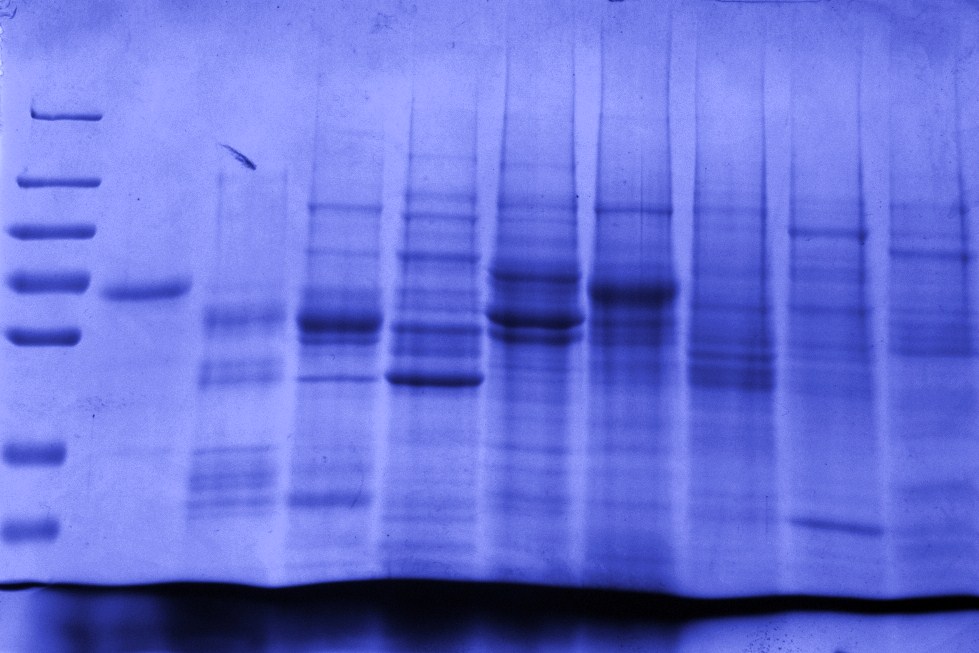

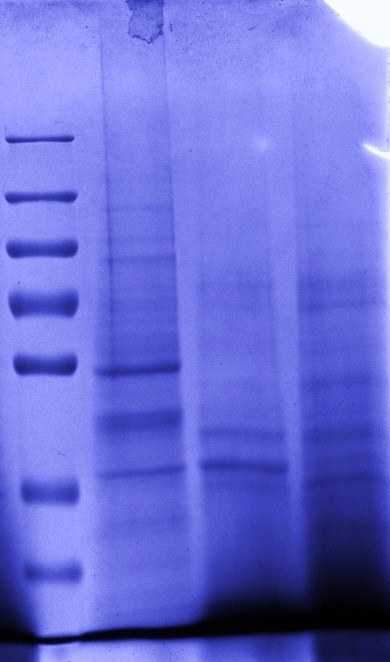


m 1 2 3 4 5 6 7 8 9 m 10 11 12

m 1 2 3 4 5 6 7 8 9 m 10 11 12

Untreated Rin-5F cells (control)

IAPP treated Rin-5F cells

***SI Fig. 1*** SDS-PAGE analysis of the OFFGEL^TM^ fractions of untreated and IAPP treated Rin-5F cells from 3 independent experiments (Ex1, Ex2 and Ex3). Numbers 1-12 represent the corresponding fractions of 1-12 from the OFFGEL^TM^ electrophoresis. The letter ‘m’ is an abbreviation for molecular weight markers (250, 130, 95, 72, 55, 36 and 28 kDa).
