# Supplementary material for: Effect of IAPP on the proteome of cultured Rin-5F cells
Source: BMC Biochem. 2018 Nov 12;19:9. doi: 10.1186/s12858-018-0099-3 (PMC6233276; doi:10.1186/s12858-018-0099-3)
Supplement: Supplementary file 3 — Table S2. Protein Mass Spectrometry Data Table Descriptors. (DOCX 12 kb) [file 12858_2018_99_MOESM3_ESM.docx]

**SI Table 2.** Protein Mass Spectrometry Data Table Descriptors

Accession UniProtKB Accession Number, the unique identifier of an entry in the UniProtKB sequence database.

Description Protein name

mW (Da) Molecular Weight of protein in Daltons

pI (pH) pH at which protein has zero charnge

PLGS Score Waters ProteinLynx Global Server score. A statistical measure of peptide assignment accuracy.

Peptides Number of peptides identified for protein after tryptic digest

Theoretical Peptides Ideal number of peptides detected for protein after tryptic digest

Coverage (%) % of total sequence included in detected peptides

Precursor RMS Mass Error (ppm) Mean accuracy in masses of precursor ions in parts per million

Products Number of product peptides detected after MS/MS

Products RMS Mass Error (ppm) Mean accuracy in masses of product ions in parts per million

Products RMS RT Error (min) Error in elution time – we can delete this

Amount (fmol) Amount of protein present in femtomoles

Amount (ngrams) Amount of protein present in nanograms
