# Supplementary figures and images for: Effect of IAPP on the proteome of cultured Rin-5F cells
Source: BMC Biochem. 2018 Nov 12;19:9. doi: 10.1186/s12858-018-0099-3 (PMC6233276; doi:10.1186/s12858-018-0099-3)

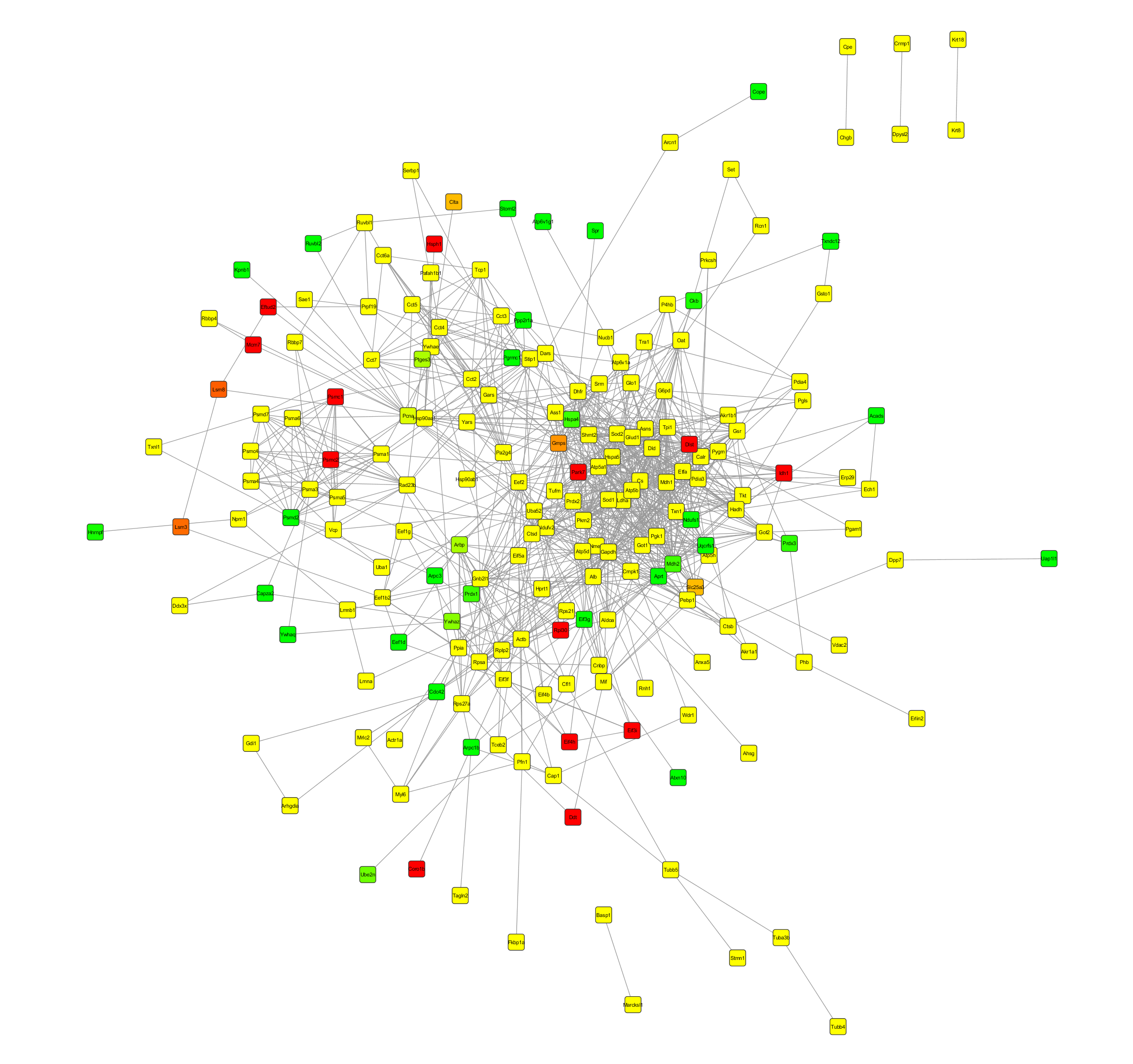

Supplement: Supplementary file 5 — Figure S2. Protein-Protein Interaction Network; Up-regulated proteins are colour coded by red, down-regulated by green and unchanged by yellow. (TIF 877 kb) [file 12858_2018_99_MOESM5_ESM.tif]
